# Enzyme activities of α-glucosidase in Japanese neonates with pseudodeficiency alleles

**DOI:** 10.1016/j.ymgmr.2017.06.007

**Published:** 2017-07-07

**Authors:** Ryuichi Mashima, Torayuki Okuyama

**Affiliations:** Department of Clinical Laboratory Medicine, National Center for Child Health and Development, 2-10-1 Okura, Setagaya-ku, Tokyo 157-8535, Japan

## Abstract

Lysosomal storage disorders (LSDs) are caused by defective enzyme activities in lysosomes, characterized by the accumulation of sphingolipids, glycolipids, oligosaccharides, mucopolysaccharides, the oxidation products of cholesterol, and other biological substances. A growing number of clinical studies have suggested the enhanced efficacy of existing therapies, including enzyme replacement therapy, which is effective when it is initiated during the presymptomatic period. Thus, the identification of disease-affected individuals by newborn screening has been considered an effective platform. Previous studies have suggested that the discrimination of infantile-onset Pompe disease (IOPD) requires multi-step examination of GAA enzyme activity using the fluorometric technique. In sharp contrast, the MS/MS-based technique can identify the population of IOPD and the pseudodeficiency alleles of the GAA enzyme [Liao HC et al. Clin Chem (2017) in press; doi: http://dx.doi.org/10.1373/clinchem.2016.269027]. To determine whether MS/MS-based assay can identify these two populations in Japanese neonates, we first performed a validation study of this assay using flow-injection analysis (FIA)-MS/MS and liquid chromatography (LC)-MS/MS followed by examination of GAA enzyme activity in our population. By minimizing the effect of substrate-derived in-source decomposition products, the activities of 6 LSD enzymes were quantified in FIA-MS/MS and LC-MS/MS. The mean value of GAA activity with IOPD, pseudodeficiency alleles, and healthy controls by FIA-MS/MS were 1.0 ± 0.3 μmol/h/L (max, 1.3; min, 0.7; median, 1.2; *n* = 3), 2.7 ± 0.7 μmol/h/L (max, 4.5; min, 1.5; median, 2.5; *n* = 19), and 12.9 ± 5.4 μmol/h/L (max, 29.6; min, 2.5; median, 11.0; *n* = 83), respectively. These results suggest that the population of GAA with pseudodeficiency alleles has approximately 20% of GAA enzyme activity compared to controls, providing the preliminary evidence to estimate the cut-off values in the Japanese population using this technique.

## 1 Introduction

Lysosomal storage disorders (LSDs) are caused by defective enzyme activities in lysosomes, characterized by the accumulation of oligosaccharides, glycolipids, mucopolysaccharides, sphingolipids, the oxidation products of cholesterol, and other biological substances [1], [2]. Although the prevalence of these diseases is rare, many revolutionary therapies have been developed. Due to its high effectiveness, enzyme replacement therapy is one of the most well-appreciated treatments for these disorders. Accumulating evidence has suggested that the treatment for LSDs has, in general, maximal benefit to the disease-affected individuals. To achieve this benefit, newborn screening for LSD has been considered a promising platform [3].

A tandem mass spectrometry (MS/MS)-based enzyme assay for LSDs was first reported in 2004 ([4], reviewed in [5]). Since then, several newborn screening programs have been performed based on this assay [6], [7], [8]. The advantage of this method is strongly associated with the inclusion of individual, internal standards for multiple enzymes in each assay reaction, which enhances the assay's accuracy dramatically. Furthermore, this MS/MS-based assay usually gives lower background compared to fluorometric assay because the accumulating enzyme reaction product of each reaction can be selectively quantified using the mixed reaction monitoring mode. These two advantages provide MS/MS-based assay technique with a wider range of enzyme activity quantification. The analytical range is a measure defined as the ratio of enzyme activity of healthy controls to that of blank [9]. This analytical range is closely associated with the lowest limit of enzyme activity quantification [8], [9], [10]. The analytical ranges for the MS/MS-based method are normally 3- to 10-fold higher than those of fluorometric assay, suggesting that the disease-affected population can be directly identified by enzyme activity using a DBS, rather than a leukocyte concentrate. Based on the abovementioned advantage, the number of diseases by which enzyme activity can be quantified using MS/MS-based methodology is now expanding [9].

In Pompe disease, at least two types of phenotype have been established [11]. One is an infantile-onset Pompe disease (IOPD), which limits the life expectancy of the affected individual by two years when the appropriate treatment has not been provided. The major manifestations include cardiomegaly, hypotonia, muscular weakness, and hepatomegaly. The other phenotype is late-onset Pompe disease (LOPD), which is usually recognized in adults because of the elevated enzyme activity of creatinine kinase [12], [13]. For newborn screening, the population of IOPD must be identified from that of healthy subjects. It is known that there is a minor, but distinct sub-population with low enzyme activity associated with the pseudodeficiency alleles of the c.1726G>A (p.G576S) mutation of α-glucosidase (GAA) [14], [15]. This mutation has been found in the populations of Asian countries such as Japan and Taiwan. Overall, the mutation occurs in ~ 3% of the total Asian population [14], [16], [17]. Importantly, a recent study clearly demonstrated that a population with IOPD/LOPD has approximately 1% or even less enzyme activity when the MS/MS-based method was employed, whereas the sub-population of the GAA pseudodeficiency alleles has nearly 5–10% enzyme activity [18]. In this study, we first validated this assay using flow-injection analysis (FIA)-MS/MS and liquid chromatography (LC)-MS/MS, respectively. Then, we further demonstrated the levels of GAA enzyme activity in populations with IOPD and the GAA pseudodeficiency alleles using this technique.

## 2 Experimental procedure

### 2.1 Reagents

The substrates and internal standards for α-glucosidase (GAA), α-galactosidase A (GLA), α-L-iduronidase (IDUA), glucocerebrosidase (ABG), acid sphingomyelinase (ASM), and galactosylceramidase (GALC) were purchased from PerkinElmer (Waltham, MA). Acetonitrile and methanol were purchased from Fischer Scientific (Tokyo, Japan). Deionized water was obtained through a Milli-Q water system from Millipore (Milford, MA). Formic acid was purchased from Kanto Chemical (Tokyo, Japan). Ammonium acetate and ethyl acetate were purchased from Wako Pure Chemicals (Tokyo, Japan). The other reagents used in this study were of the highest grade commercially available.

### 2.2 Approval by institutional research ethics board

This study was approved by the Research Ethics Board of the National Center for Child Health and Development (Tokyo, Japan).

### 2.3 Dried blood spot (DBS) specimens for quality control (QC)

The DBSs for QC were kindly provided by Dr. Jonathan Rehnberg at Diagnostics Division, PerkinElmer (Turku, Finland) and Dr. Anna Potier at Diagnostics Division, PerkinElmer (Waltham, MA).

### 2.4 Determination of enzyme activities of six LSDs by LC-MS/MS

The preparation of enzyme reaction used to determine 6 LSD enzyme activities has been previously reported [19]. In brief, the enzymes were extracted from the DBSs (3 mm in diameter) using an automated puncher (model 1296–071 DELFIA® Dried blood Spot Punch, PerkinElmer) and reacted with substrates in a buffer (30 μL) for 20 h at 37 °C in a 96-well plate. The concentrations of the substrates and internal standards were as follows: GAA, 0.35 mM, 24 μM; GLA, 1.2 mM, 24 μM; IDUA, 0.25 mM, 15 μM; ABG, 0.5 mM, 20 μM; ASM, 0.75 mM, 15 μM; and GALC, 0.85 mM, 10 μM. To terminate the reaction, a mixture of ethyl acetate/methanol (50/50, 100 μL) was added. This reaction mixture was then transferred to a 96-well deep plate, and ethyl acetate (400 μL) and water (200 μL) were added. After mixing and centrifugation, the supernatant (75 μL) was transferred to a 96-well shallow plate. This organic solution was then dried under an N_2_ stream and reconstituted with the mobile phase (150 μL, H_2_O/CH_3_CN/formic acid = 20/80/0.002).

### 2.5 Analytical procedure

Flow-injection analysis (FIA)-MS/MS: The sample solution was delivered into a LCMS8030plus MS/MS spectrometer (Shimadzu, Kyoto, Japan) using an HPLC system Nexera (Shimadzu) at a flow rate of 0.1 mL/min. Typically, an aliquot (1–5 μL) was injected onto FIA-MS/MS. MS/MS conditions were optimized, as described in Supplementary Procedure.

Liquid chromatography (LC)-MS/MS: The levels of enzyme reaction products were analyzed using the same LCMS8030plus mass spectrometer and Nexera HPLC system. We used a MonoTower C18 (3 × 50 mm) or an InertSustainSwift C18 (2.1 × 30 mm, 3 μm) analytical column purchased from GL Sciences (Tokyo, Japan). Details of instrumental parameters are available in Supplementary Tables 1–5.

### 2.6 Determination of enzyme activities in DBSs

The activity of each enzyme was determined by examining the accumulation of the reaction product using the corresponding internal standard in μmol/h/L of blood, where each 3-mm DBS punch contained 3.1 μL of blood. Both the positive mode of electrospray ionization and multiple-reaction monitoring mode were used to quantify the enzyme reaction products.

### 2.7 Identification of GAA c.1726G>A alleles

GAA enzyme activity was screened using 4MU as a substrate in the presence of acarbose [15]. Then, the specimen with low enzyme activity was further examined through the PCR-based technique using the sequences 5′-AGG GAG GGC ACC TTG GAG CCT G-3′ and 5′-GGG AGG CGA TGG CTT CGG TCA AG-3′ as the forward and reverse primers, respectively [15]. All individuals with pseudodeficiency alleles of GAA in this study had homozygous c.1726G>A alleles.

## 3 Results

To validate GAA assay using FIA-MS/MS- and LC-MS/MS-based protocols, we first examined the CV values of GAA activity for intraday and interday assays. As shown, the values of intraday CV (%) values in FIA-MS/MS and LC-MS/MS were 4.5 and 8.4%, respectively, when methanol was used as a mobile phase, whereas those of interday CV (%) values in FIA-MS/MS and LC-MS/MS were 8.8 and 13.9%, respectively (Table 1). Under these assay conditions, the values of the analytical range in FIA-MS/MS and LC-MS/MS were 76 and 76, respectively. These results were consistent when acetonitrile was chosen for the mobile phase (Table 1). In addition to GAA, the other enzymes such as GLA, IDUA, ABG, ASM, and GALC exhibited similar levels of intra- and inter-day CV (%) values. The levels of enzyme activity of GAA in FIA-MS/MS and LC-MS/MS were comparable (Table 2). Although the analytical range in GAA using FIA-MS/MS and LC-MS/MS resulted similar, these values in ABG, ASM, and GALC were increased when LC-MS/MS was used. The enzyme activities of ABG and ASM required optimization of MS/MS settings due to high in-source decomposition of corresponding substrates when FIA-MS/MS was performed (Supplementary Fig. S1, Supplementary Fig. S2; Supplementary Table 2).

**Table 1:** Intraday and interday assay precision for multiple analyses of control samples.

Method	Mode of elution	Solvent		Intraday CV (%)							Interday CV (%)					
			GAA	GLA	IDUA	ABG	ASM	GALC	*n*	GAA	GLA	IDUA	ABG	ASM	GALC	*n*
FIA-MS/MS	Isocratic	CH_3_CN	9.1	24.7	21.6	1.5	2.5	3.0	5	14.4	14.2	19.0	14.5	19.9	18.0	4
FIA-MS/MS	Isocratic	CH_3_OH	4.5	9.0	15.2	2.9	5.6	2.2	5	8.8	12.9	24.6	12.6	18.9	12.4	4
LC-MS/MS	Isocratic	CH_3_CN	3.8	3.4	6.1	2.4	0.8	2.4	5	11.9	16.6	13.7	24.0	27.8	16.5	4
LC-MS/MS	Gradient	CH_3_OH	8.4	2.8	7.6	7.8	7.3	5.6	5	13.9	19.1	19.8	10.6	6.0	12.3	4

CH_3_CN, acetonitrile; CH_3_OH, methanol; CV, coefficient of variation.

**Table 2:** The enzyme activity in DBS of a healthy individual with its analytical range.

Enzyme	Method	Mode of elution	Run time (min)	Solvent	Enzyme activity (μmol/h/L blood)	Analytical range^a^ (−)
GAA	FIA-MS/MS	Isocratic	2	Acetonitrile	6.6	76
	FIA-MS/MS	Isocratic	2	Methanol	7.3	76
	LC-MS/MS	Isocratic	5	Acetonitrile	5.5	76
	LC-MS/MS	Gradient	7	Methanol	6.3	62

GLA	FIA-MS/MS	Isocratic	2	Acetonitrile	2.8	38
	FIA-MS/MS	Isocratic	2	Methanol	3.4	42
	LC-MS/MS	Isocratic	5	Acetonitrile	4.9	22
	LC-MS/MS	Gradient	7	Methanol	4.0	38

IDUA	FIA-MS/MS	Isocratic	2	Acetonitrile	2.9	36
	FIA-MS/MS	Isocratic	2	Methanol	3.0	36
	LC-MS/MS	Isocratic	5	Acetonitrile	3.1	26
	LC-MS/MS	Gradient	7	Methanol	3.0	27

ABG	FIA-MS/MS	Isocratic	2	Acetonitrile	4.6	12
	FIA-MS/MS	Isocratic	2	Methanol	4.1	10
	LC-MS/MS	Isocratic	5	Acetonitrile	3.2	64
	LC-MS/MS	Gradient	7	Methanol	3.3	162

ASM	FIA-MS/MS	Isocratic	2	Acetonitrile	1.4	14
	FIA-MS/MS	Isocratic	2	Methanol	1.5	16
	LC-MS/MS	Isocratic	5	Acetonitrile	1.0	100
	LC-MS/MS	Gradient	7	Methanol	1.8	173

GALC	FIA-MS/MS	Isocratic	2	Acetonitrile	2.1	7
	FIA-MS/MS	Isocratic	2	Methanol	2.4	5
	LC-MS/MS	Isocratic	5	Acetonitrile	1.7	64
	LC-MS/MS	Gradient	7	Methanol	2.0	56

NA, not available. ND, not determined.

a Analytical range is defined by the enzyme activity in a healthy adult DBS divided by that in a filter paper [8], [10].

**Fig. 1:**
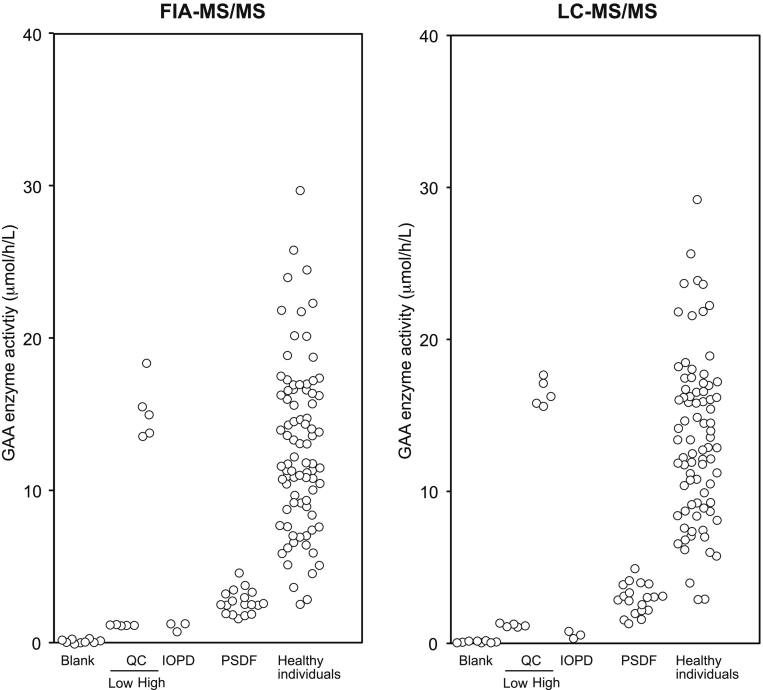
Determined GAA enzyme activity using two MS/MS-based assays. (A) GAA activity measured using FIA-MS/MS assay. (B) GAA activity measured using LC-MS/MS-based assay. Note that both assays gave almost identical results. QC DBS was provided by PerkinElmer (Turku, Finland). The numbers of samples were as follows: filter paper blank (*n* = 9); QC DBS with low activity of normal level (*n* = 5); QC DBS with high activity of normal level (*n* = 5); individuals with IOPD (*n* = 3); the GAA pseudodeficiency alleles (PSDF, *n* = 19); and healthy controls (*n* = 83), respectively. Methanol was used as the solvent.

Based on the results of this validation study, we determined whether the enzyme activity of individuals with IOPD and the GAA pseudodeficiency alleles may be distinguished using any of our assay conditions. To prove this, we first examined whether the GAA enzyme activity in DBS provided for QC by PerkinElmer (Turku) may be quantified by our procedure with minimal assay error. In fact, the GAA activities of this QC DBS in different lots (#6276967, #6276958, #6276911 from Lot# 01209–53 and #1122398 from Lot# 01193-54) were almost identical in the FIA-MS/MS (left) and LC-MS/MS (right) methods (Fig. 1). Consistently, the GAA enzyme activities expressed as mean ± SD in μmol/h/L blood (max, min, median, *n*) of individuals with IOPD, pseudodeficiency alleles, and healthy individuals were 1.0 ± 0.3 μmol/h/L blood (max, 1.3; min, 0.7; median, 1.2; *n* = 3), 2.7 ± 0.7 μmol/h/L blood (max, 4.5; min, 1.5; median, 2.5; *n* = 19), and 12.9 ± 5.4 μmol/h/L blood (max, 29.6; min, 2.5; median, 11.0; *n* = 83) using FIA-MS/MS assay, whereas those using LC-MS/MS were 0.5 ± 0.1 μmol/h/L blood (max, 0.6; min, 0.4; median, 0.6; *n* = 3), 2.9 ± 1.0 μmol/h/L blood (max, 4.8; min, 1.3; median, 3.0; *n* = 19), and 13.5 ± 5.3 μmol/h/L blood (max, 29.2; min, 2.8; median, 11.9; *n* = 83), respectively (Fig. 1 and Table 3). In both cases, the upper levels of GAA enzyme activity in IOPD were lower than the lowest levels of individuals with pseudodeficiency alleles. Importantly, the difference was much clearer when LC-MS/MS was used. These results indicated that both FIA-MS/MS- and LC-MS/MS-based assays identify whether individuals have IOPD.

**Table 3:** Enzyme activity of individuals with IOPD and GAA pseudodeficiency alleles using FIA-MS/MS.

Sample	ID	Enzyme activity^a^ (μmol/h/L blood)	Relative activity (% of healthy control)
Blank (*n* = 9)	NA	0.1 ± 0.0^b^	0.8
Healthy control (*n* = 83)	NA	12.9 ± 5.4^b^	100
IOPD^c^	1	1.2	9.4
	2	1.3	9.7
	3	0.7	5.2
GAA pseudodeficiency	1	1.8	14.1
	2	2.5	19.7
	3	2.9	22.8
	4	1.9	15.0
	5	1.8	14.0
	6	4.5	35.1
	7	3.3	25.5
	8	3.7	28.6
	9	2.6	20.1
	10	2.6	20.5
	11	2.7	20.8
	12	2.5	19.7
	13	2.4	18.9
	14	3.2	24.8
	15	1.5	12.0
	16	1.8	14.5
	17	2.5	19.3
	18	2.5	19.3
	19	3.4	26.4

a Enzyme activity was determined using methanol as a mobile phase.

b Mean ± SD.

c IOPD, infantile-onset Pompe disease.

## 4 Discussion

GAA pseudodeficiency allele c.1736G>A (p.G576S) is a well-known genetic mutation found in Asian countries [14], [15]. The homology-based structural prediction of GAA p.G576S revealed a small alteration, leading to 15% and 11% normal enzyme activity for an artificial substrate and glycogen, respectively [20]. There was no accumulation of glycogen in organs, including skeletal muscle, in these individuals, demonstrating that even this attenuated activity can maintain cellular homeostasis of glycogen storage in the cells. An earlier study identified that 3% of the Taiwanese population carry this genetic mutation [14]. Two Japanese studies that consistently support these results [15], [16]. Compared to these Asian areas, the frequency of the GAA c.1736G>A pseudodeficiency allele in European and Sub-Saharan/African population are rare [21]. In fact, the identification of a sub-population with the GAA pseudodeficiency was problematic using fluorometric assay [15]. A recent study using MS/MS-based assay clearly addressed this issue because these two populations can be identified only when the MS/MS-based technique is employed [18]. Our results consistently support this observation and further provide an additional example that this MS/MS-based assay procedure identified this sub-population of individuals with the GAA pseudodeficiency alleles.

A pseudodeficiency allele is a mutation that generates an altered protein product but does not cause disease. Among LSDs, it is well-known that there are some examples of pseudodeficiency alleles found in several disorders including Pompe disease [22]. For example, in mucopolysaccharidosis type I, four pseudodeficiency IDUA alleles were recently identified (p.A79T, p.H82Q, p.D223N, and p.V322E) in newborn screening in Missouri [23]. In Fabry disease, an earlier study reported that p.D313Y is a GLA pseudodeficiency mutant with minimal alteration of enzyme structure [24]. Furthermore, there are many reports on the pseudodeficiency alleles on arylsulfatase A (ARSA), an enzyme responsible for metachromatic leukodystrophy [25], [26]. In the case of ARSA, subsequent evidences have shown that there are many pseudodeficiency mutations that are not limited to a restricted area but are global [27], [28]. In fact, its number is still increasing these days [29], [30]. Based on this fact, although the method for the screening of metachromatic leukodystrophy by quantifying sulfatides has been reported, further efforts have been made to improve this assay with reasonable accuracy for screening [31], [32].

The 6-plex LSD assay has been developed by a research group directed by Professor Michael H Gelb in 2004 ([4], reviewed in [5]). Since then, a growing number of newborn screening programs adopted this methodology globally. Initially, this assay has been designed to perform newborn screening of 6 LSD enzymes, including GAA, GLA, IDUA, ABG, ASM, and GALC. Furthermore, the activity of other LSD enzymes such as iduonate-2-sulfatase for MPS II, *N*-acetylgalactosamine-6-sulfatase for MPS IVA, *N*-acetylgalactosamine-4-sulfatase for MPS VI, α-*N*-acetylglucosainidase for MPS IIIB, and lysosomal β-glucuronidase for MPS VII has been developed [9]. Due to the expansion of the MS/MS-based assay, there are many variations of assay procedure [5], [8], [33]. In some cases, this assay has been performed with C26:0-lysophosphatidylcholine [33]. Particular, this study examined the enzyme activity of two populations, such as healthy and disease-affected individuals. Thus, our data provides the evidence that the population of individuals with pseudodeficiency of GAA enzyme can be identified using this platform.

In conclusion, we provided evidences that the MS/MS-based 6-LSD enzyme assay can identify the population of individuals with GAA pseudodeficiency alleles. To achieve this, assay validation must be properly performed. Our results also show that this assay can be performed in any facility with a standard MS/MS instrument, suggesting a potential expansion of this assay in many screening facilities.

The following are the supplementary data related to this article.

Supplementary procedure Image 1

Supplementary Fig. S1 Changes in peak area by altering temperatures of desolvation line and heatblock. Supplementary Fig. S1

Supplementary Fig. S2 Changes in peak area by altering the flow rate of nebulizer gas and drying gas. Supplementary Fig. S2
