# Supplementary material for: Enzyme activities of α-glucosidase in Japanese neonates with pseudodeficiency alleles
Source: Mol Genet Metab Rep. 2017 Jul 7;12:110–4. doi: 10.1016/j.ymgmr.2017.06.007 (PMC5503834; doi:10.1016/j.ymgmr.2017.06.007)
Supplement: Supplementary file 1 — Supplementary procedure [file mmc1.docx]

**Supplementary Procedure**

**Optimization for the reduction of in-source decomposition products from substrates of ABG and ASM in FIA-MS/MS 6-plex assay**

To minimize the artificially accumulating decomposition products from substrates of ABG and ASM enzyme reactions, we carefully examined 4 parameters of the MS detector, such as desolvation temperature (°C), heatblock temperature (°C), flow rate of nebulizer gas (L/h), and flow rate of drying gas (L/h). First, the peak areas of artificially generating ABG product through in-source decomposition (ABG_P_ISD_) and of ABG internal standard (ABG_IS) were quantified under various desolvation temperature and heatblock temperature to find out smaller level of ABG_P_ISD_/ABG_IS (%) (Supplementary Fig. 1). Based on this result, we were able to find out that desolvation temperature needed to be below 250°C. Then, we further investigated the flow rates of nebulizer gas and drying gas under various conditions (Supplementary Fig. 2). When desolvation temperature and heatblock temperature were set at 120°C and 150°C, respectively, any combination of the flow rate of nebulizer gas (0.5 - 3 L/h) and of drying gas (3 - 20 L/h) gave lower ABG_P_ISD_/ABG_IS (%) (Supplementary Fig. 2). Note that the levels of GAA_P_ISD_/ABG_IS (%) remained consistently low under our experimental conditions.

**Supplementary Table 1**

FIA methods for the measurement of enzyme activities for 6 LSDs.

| Solvents | CH_3_CN | CH_3_OH |
| --- | --- | --- |
| Temperature | 40°C | 40°C |
| Wash solvent | CH_3_OH | CH_3_OH |
| Mobile phase | A: 0.2% formic acid in H_2_O  B: CH_3_CN | A: 5 mM NH_4_OAc in H_2_O  B: 0.1% FA in CH_3_OH |
| B (%) | 80 | 80 |
| Flow rate | 0.1 mL/min | 0.1 mL/min |
| Injection volume | 1-5 μL | 1-5 μL |
| Injection mode | Direct injection | Direct injection |
| Autosampler | 4°C | 4°C |

NH_4_OAc, ammonium acetate.

**Supplementary Table 2**

Instrument parameters for the quantification of substrates, internal standards and products of 6 LSD enzyme activities using MS/MS.

| Method | FIA-MS/MS | LC-MS/MS |
| --- | --- | --- |
| Interface | ESI | ESI |
| Polarity | Positive | Positive |
| Capillary voltage | 2.0 kV | 2.0 kV |
| Heatblock temperature | 150°C | 400°C |
| Desolvation temperature | 120°C | 250°C |
| Flow rate of nebulizing gas | 3 L/min | 3 L/min |
| Flow rate of drying gas | 15 L/min | 15 L/min |
| Analyzing mode | MRM | MRM |
| Scan time | 20 ms | 20 ms |
| Data format | Centroid | Centroid |

ESI, electrospray ionization.

**Supplementary Table 3**

Product ion, precursor ion, cone voltage and collision energy for the fragmentation of substrates, products and internal standards.

|  | Precursor ion | Product ion | Q1 Pre Bias | CE | Q3 Pre Bias |
| --- | --- | --- | --- | --- | --- |
|  | (m/z) | (m/z) | (V) | (V) | (V) |
| GAA_IS | 503.1 | 403.1 | -34 | -16 | -18 |
| GAA_P | 498.1 | 398.1 | -34 | -16 | -18 |
| GLA_IS | 489.1 | 389.05 | -18 | -15 | -25 |
| GLA_P | 484.1 | 384.05 | -18 | -15 | -25 |
| IDUA_IS | 431.1 | 322.05 | -28 | -16 | -21 |
| IDUA_P | 426.1 | 317.05 | -28 | -16 | -21 |
| ABG_IS | 391.2 | 271.15 | -27 | -22 | -27 |
| ABG_P | 384.4 | 264.15 | -27 | -22 | -27 |
| ASM_IS | 405.2 | 264.15 | -28 | -21 | -17 |
| ASM_P | 398.2 | 264.15 | -28 | -21 | -17 |
| GALC_IS | 417.2 | 264.1 | -29 | -21 | -17 |
| GALC_P | 412.2 | 264.1 | -29 | -21 | -17 |
| GAA_S | 660.2 | 560.15 | -22 | -16 | -26 |
| GLA_S | 646.2 | 546.15 | -22 | -18 | -26 |
| IDUA_S | 602.1 | 317.05 | -38 | -21 | -21 |
| ABG_S | 546.1 | 264.3 | -36 | -32 | -26 |
| ASM_S | 563.2 | 184.0 | -36 | -22 | -30 |
| GALC_S | 574.2 | 264.05 | -38 | -28 | -17 |

GAA, α-glucosidase; GLA, α-galactosidase A ; IDUA, α-L-iduronidase; ABG, glucocerebrosidase; ASM, acid sphingomyelinase; GALC, galactosylceramidase.

**Supplementary Table 4**

Retention times of S/IS for 6 LSD enzymes.

| Column | MonoTower C18 | | InertStainSwift C18 |
| --- | --- | --- | --- |
| Inner diameter (mm) | 3 | | 2.1 |
| Length (mm) | 50 | | 30 |
| Particle (μm) | NA | | 3 |
| Solvent A: | 0.02% Formic acid in H_2_O | 5 mM NH_4_OAc in H_2_O | 5 mM NH_4_OAc in H_2_O |
| Solvent B: | Acetonitrile | 0.1% Formic acid in methanol | 0.1% Formic acid in methanol |
|  | Retention time (min) | | |
| GAA_IS | 0.584 | 0.649 | 1.442 |
| GAA_S | 0.557 | 0.601 | 2.072 |
| GLA_IS | 0.566 | 0.624 | 2.146 |
| GLA_S | 0.538 | 0.583 | 1.751 |
| IDUA_IS | 0.529 | 0.554 | 0.760 |
| IDUA_S | 0.557 | 0.503 | 0.608 |
| ABG_IS | 1.661 | 4.854 | 4.651 |
| ABG_S | 0.985 | 3.883 | 4.286 |
| ASM_IS | 2.035 | 6.309 | 5.113 |
| ASM_S | 1.113 | 5.229 | 4.643 |
| GALC_IS | 2.482 | 8.444 | 5.674 |
| GALC_S | 1.286 | 5.584 | 5.136 |

**Supplementary Table 5**

Retention times of S/IS for 6 LSD enzymes using gradient elution.

| Run | #1 | #2 | #3 | #4 | #5 | #6 | #7 | #8 | #9 | #10 | #11 |
| --- | --- | --- | --- | --- | --- | --- | --- | --- | --- | --- | --- |
|  |  |  |  |  |  |  |  |  |  |  |  |
| Compound | Retention time | | | | | | | | | | |
|  | min | | | | | | | | | | |
| GAA_IS | 0.491 | 1.382 | 2.345 | 2.345 | 2.290 | 2.018 | 1.981 | 2.018 | 2.018 | 2.072 | 1.442 |
| GAA_S | 0.491 | 0.981 | 2.003 | 2.003 | 2.015 | 1.888 | 1.834 | 1.852 | 1.852 | 1.867 | 2.072 |
| GLA_IS | 0.512 | 1.092 | 2.108 | 2.126 | 2.072 | 1.909 | 1.89 | 1.909 | 1.909 | 1.945 | 2.146 |
| GLA_S | 0.491 | 0.781 | 1.708 | 1.745 | 1.708 | 1.671 | 1.634 | 1.671 | 1.652 | 1.674 | 1.751 |
| IDUA_IS | 0.445 | 0.527 | 0.746 | 0.764 | 0.729 | 0.746 | 0.746 | 0.746 | 0.764 | 0.723 | 0.760 |
| IDUA_S | 0.399 | 0.442 | 0.502 | 0.501 | 0.502 | 0.513 | 0.510 | 0.501 | 0.502 | 0.551 | 0.608 |
| ABG_IS | 2.011 | 3.484 | 3.768 | 3.857 | 3.822 | 3.822 | 2.987 | 3.573 | 3.449 | 3.822 | 4.651 |
| ABG_S | 1.671 | 3.413 | 3.667 | 3.757 | 3.649 | 3.503 | 2.832 | 3.34 | 3.231 | 3.518 | 4.286 |
| ASM_IS | 2.420 | 3.627 | 3.84 | 3.964 | 3.982 | 4.266 | 3.165 | 3.858 | 3.627 | 4.292 | 5.113 |
| ASM_S | 2.043 | 3.489 | 3.767 | 3.842 | 3.823 | 3.842 | 2.988 | 3.600 | 3.434 | 3.851 | 4.643 |
| GALC_IS | 2.996 | 3.681 | 3.930 | 4.054 | 4.196 | 4.786 | 3.379 | 4.16 | 3.823 | 4.829 | 5.674 |
| GALC_S | 2.434 | 3.601 | 3.842 | 3.953 | 3.972 | 4.267 | 3.16 | 3.849 | 3.619 | 4.222 | 5.136 |
|  |  |  |  |  |  |  |  |  |  |  |  |
|  |  |  |  |  |  |  |  |  |  |  |  |
|  | Gradient program | | | | | | | | | | |
|  | Time (min): B (%) | | | | | | | | | | |
|  | 0.01: 80 | 0.01: 60 | 0.01: 50 | 0.01: 50 | 0.01: 50 | 0.01: 50 | 0.01: 50 | 0.01: 50 | 0.01: 50 | 0.01: 50 | 0.01: 50 |
|  | 5.00: End | 0.50 60 | 0.50: 50 | 0.50: 50 | 0.50: 50 | 0.50: 50 | 0.50: 50 | 0.50: 50 | 0.50: 50 | 0.50: 50 | 0.50: 50 |
|  |  | 3.50: 100 | 3.50: 100 | 3.00: 90 | 2.50: 85 | 1.50: 80 | 1.50: 85 | 1.50: 80 | 1.50: 80 | 1.50: 80 | 2.50: 80 |
|  |  | 4.00: 100 | 4.00: 100 | 4.00: 90 | 4.00: 85 | 4.00: 80 | 4.00: 85 | 4.00: 85 | 4.00: 90 | 5.00: 85 | 6.00: 90 |
|  |  | 4.01: 60 | 4.01: 50 | 4.01: 50 | 4.01: 50 | 4.01: 50 | 4.01: 50 | 4.01: 50 | 4.01: 50 | 5.01: 50 | 6.01: 50 |
|  |  | 5.00: End | 5.00: End | 5.00: End | 5.00: End | 5.00: End | 5.00: End | 5.00: End | 5.00: End | 6.00: End | 7.00: End |

InertSustainSwift C18 (2.1 × 50 mm, 3 μm, GL Sciences, Tokyo, Japan) was used.
