# Supplementary figures and images for: Enzyme activities of α-glucosidase in Japanese neonates with pseudodeficiency alleles
Source: Mol Genet Metab Rep. 2017 Jul 7;12:110–4. doi: 10.1016/j.ymgmr.2017.06.007 (PMC5503834; doi:10.1016/j.ymgmr.2017.06.007)

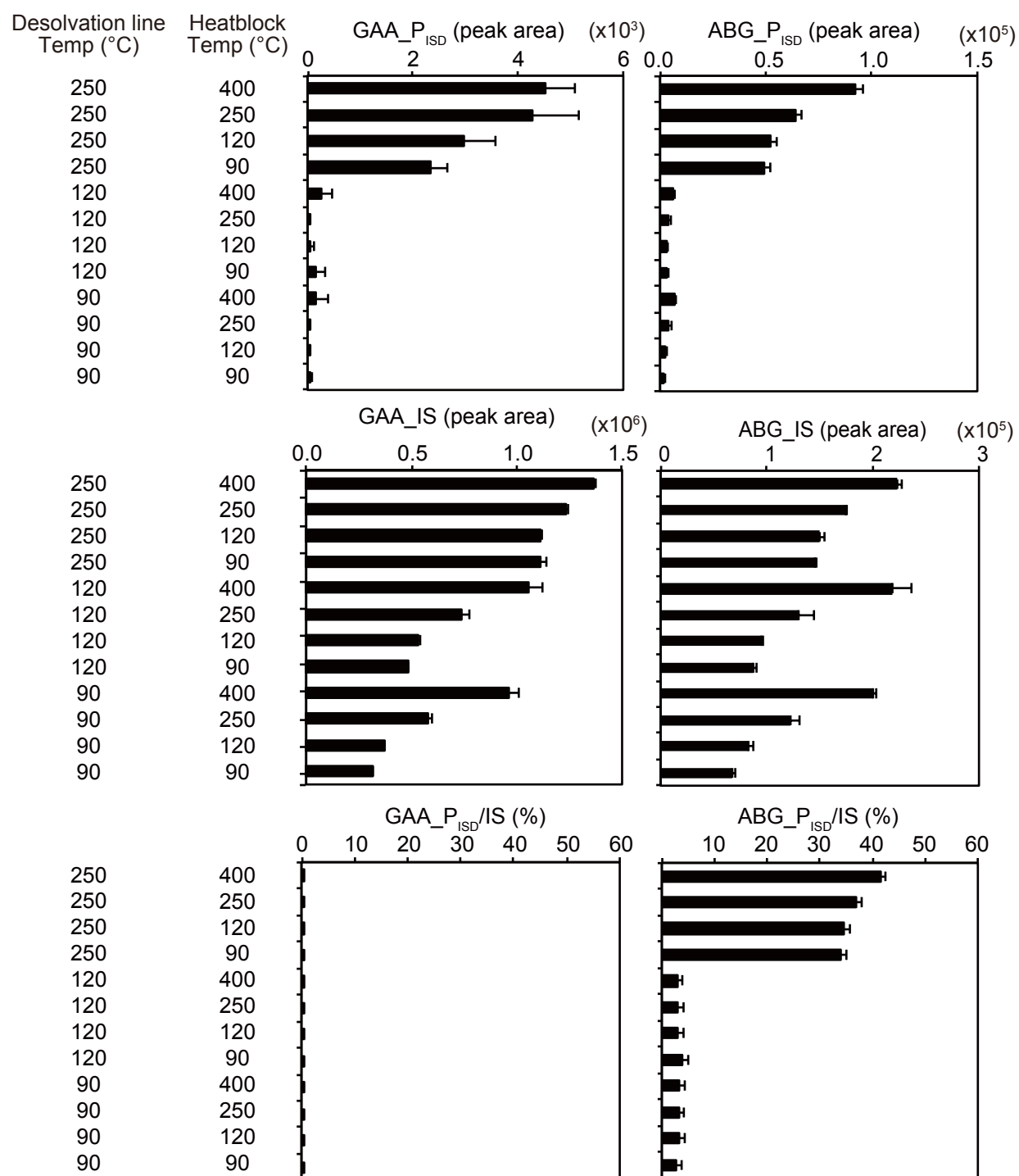

Supplementary Fig. 1  
Mashima R et al.

Supplement: Supplementary Fig. S1 — Changes in peak area by altering temperatures of desolvation line and heatblock. [file mmc2.pdf]

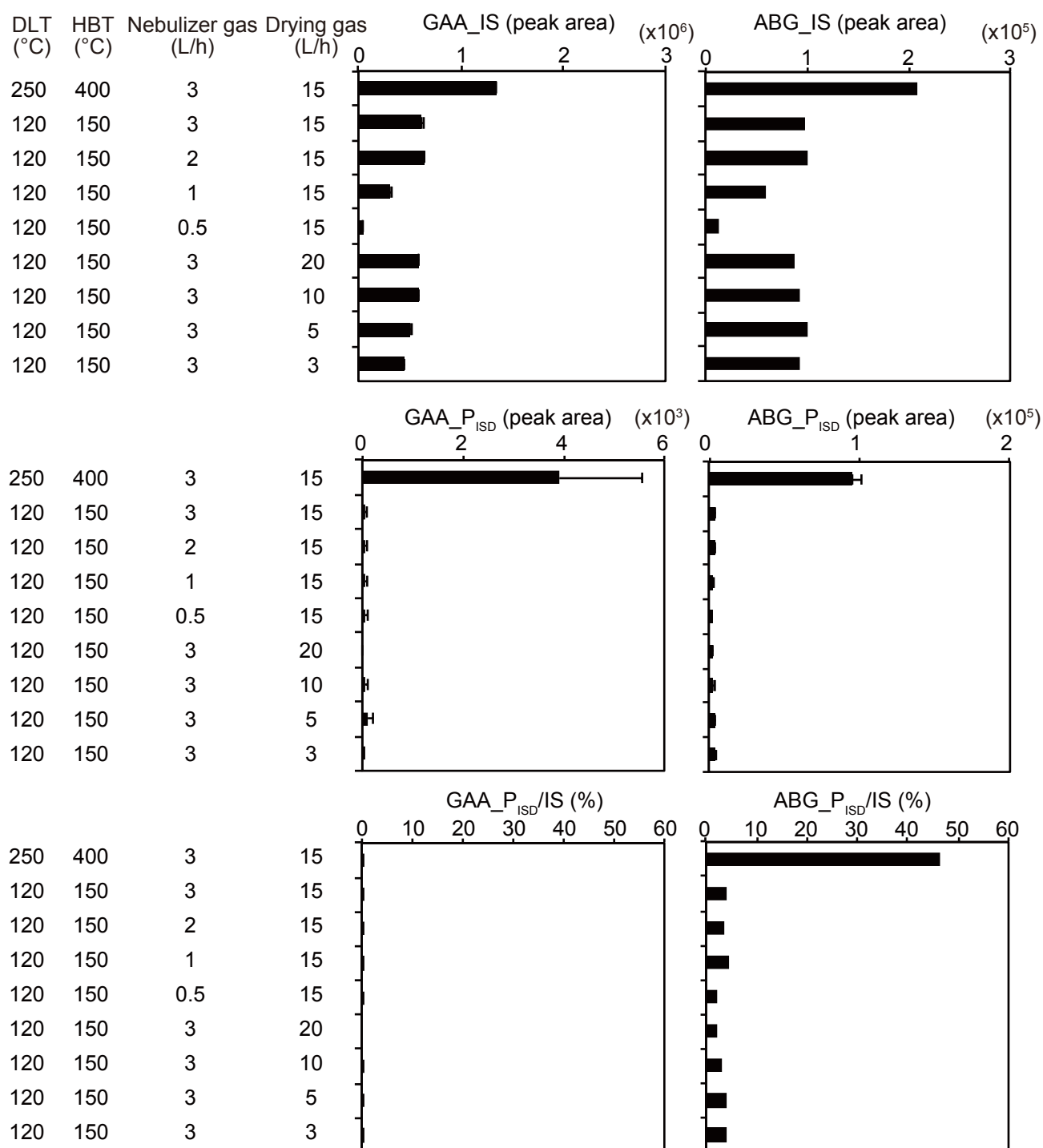

Supplementary Fig. 2  
Mashima R et al.

Supplement: Supplementary Fig. S2 — Changes in peak area by altering the flow rate of nebulizer gas and drying gas. [file mmc3.pdf]
